# Proteome profiling of *Campylobacter jejuni* 81–176 at 37 °C and 42 °C by label-free mass spectrometry

**DOI:** 10.1186/s12866-024-03348-8

**Published:** 2024-05-31

**Authors:** Annika Dreyer, Wycliffe O. Masanta, Raimond Lugert, Wolfgang Bohne, Uwe Groß, Andreas Leha, Mohammed Dakna, Christof Lenz, Andreas E. Zautner

**Affiliations:** 1 Institute for Medical Microbiology and Virology, University Medical Center Göttingen, 37075 Göttingen, Germany; 2 Department of Medical Microbiology, Maseno University Medical School, Private Bag, Maseno, 40105 Kenya; 3 Department of Medical Statistics, University Medical Center Göttingen, 37075 Göttingen, Germany; 4 Bioanalytical Mass Spectrometry Group, Max Planck Institute for Biophysical Chemistry, 37077 Göttingen, Germany; 5 Institute of Clinical Chemistry, Bioanalytics, University Medical Center Göttingen, 37075 Göttingen, Germany; 6 Institute of Medical Microbiology and Hospital Hygiene, Medical Faculty, Otto-von-Guericke-University Magdeburg, Leipziger Straße 44, 39120 Magdeburg, Germany; 7 CHaMP, Center for Health and Medical Prevention, Otto-von-Guericke-University Magdeburg, 39120 Magdeburg, Germany

**Keywords:** *Campylobacter jejuni*, Temperature changes, Proteome, Mass spectrometry, DIA-MS

## Abstract

**Background:**

The main natural reservoir for *Campylobacter jejuni* is the avian intestinal tract. There, *C. jejuni* multiplies optimally at 42 °C – the avian body temperature. After infecting humans through oral intake, the bacterium encounters the lower temperature of 37 °C in the human intestinal tract. Proteome profiling by label-free mass spectrometry (DIA-MS) was performed to examine the processes which enable *C. jejuni* 81–176 to thrive at 37 °C in comparison to 42 °C. In total, four states were compared with each other: incubation for 12 h at 37 °C, for 24 h at 37 °C, for 12 h at 42 °C and 24 h at 42 °C.

**Results:**

It was shown that the proteomic changes not only according to the different incubation temperature but also to the length of the incubation period were evident when comparing 37 °C and 42 °C as well as 12 h and 24 h of incubation. Altogether, the expression of 957 proteins was quantifiable. 37.1 − 47.3% of the proteins analyzed showed significant differential regulation, with at least a 1.5-fold change in either direction (i.e. log_2_ FC ≥ 0.585 or log_2_ FC ≤ -0.585) and an FDR-adjusted *p*-value of less than 0.05. The significantly differentially expressed proteins could be arranged in 4 different clusters and 16 functional categories.

**Conclusions:**

The *C. jejuni* proteome at 42 °C is better adapted to high replication rates than that at 37 °C, which was in particular indicated by the up-regulation of proteins belonging to the functional categories “replication” (e.g. Obg, ParABS, and NapL), “DNA synthesis and repair factors” (e.g. DNA-polymerase III, DnaB, and DnaE), “lipid and carbohydrate biosynthesis” (e.g. capsular biosynthesis sugar kinase, PrsA, AccA, and AccP) and “vitamin synthesis, metabolism, cofactor biosynthesis” (e.g. MobB, BioA, and ThiE). The relative up-regulation of proteins with chaperone function (GroL, DnaK, ClpB, HslU, GroS, DnaJ, DnaJ-1, and NapD) at 37 °C in comparison to 42 °C after 12 h incubation indicates a temporary lower-temperature proteomic response. Additionally the up-regulation of factors for DNA uptake (ComEA and RecA) at 37 °C compared to 42 °C indicate a higher competence for the acquisition of extraneous DNA at human body temperature.

**Supplementary Information:**

The online version contains supplementary material available at 10.1186/s12866-024-03348-8.

## Background

*Campylobacter jejuni* is one of the most prevalent causes of gastrointestinal disease in humans worldwide [1, 2]. Since several years, it occurs more frequently than other foodborne pathogens, such as *Salmonella* species [3]. Poultry is the main source of *Campylobacter* infections [1, 2] since it is a natural part of the microbiome in poultry [4]. Campylobacteriosis in humans is characterized by severe watery or bloody diarrhea and can result in Guillain-Barré syndrome, an acute progressive neuropathy [5–7]. In the human small intestine, *C. jejuni* predominantly inhabits the jejunum [8]. Preferentially, *C. jejuni* grows at 42 °C, the avian body temperature; however, it is able to survive at temperatures that are considered exceptional for the bacterium (from < 4 °C up to 46 °C), which promotes the spreading of infections by meat products if not properly handled [9–11]. Transcriptomic changes of *C. jejuni* as a result of heat stress have been reported previously [11, 12]. In *C. coli* and *C. lari*, Riedel et al. showed the distinct transcriptomic heat shock responses of both bacteria at 46 °C via quantitative real-time PCR and whole transcriptome sequencing [11]. The results indicate differences between the species in the expression of metabolic genes besides a general stress response. Moreover, a proteomic analysis of *C. jejuni* grown at 37 °C and 42 °C was performed by Zhang et al. in 2009 via MALDI-TOF/TOF analysis. The study revealed 24 differently expressed proteins [13]. A similar approach using a combination of 2-D electrophoresis and MALDI-TOF MS was followed by Turonova and colleagues to compare exponential and stationary growth phases. Also in this analysis, only 24 differentially expressed proteins were identified [14].

When transmitted from poultry to humans, *C. jejuni* also thrives at the human body temperature of 37 °C. The detailed processes of *C. jejuni*’s colonization of the human intestinal tract and its pathogenicity are not fully understood yet. Significant changes in the bacterial proteome can be recognized by label-free data-independent acquisition mass spectrometry (DIA-MS), which provides high reliability in the quantitative analysis of proteins [15, 16]. Here, we report the proteomic changes of *C. jejuni* strain 81–176 to a temperature drop from 42 °C to 37 °C. At this, we compare two time points, after 12 h and 24 h of incubation, which correspond to the logarithmic growth phase and the stationary phase. In addition to describing the mechanisms of the host-related temperature proteomic response, we also aim here to provide a basis for the extent to which temperature and growth phase affect the proteome of *C. jejuni*.

## Results

### Growth curve of *C. jejuni* 81–176 at 37 °C and 42 °C

**Fig. 1:**
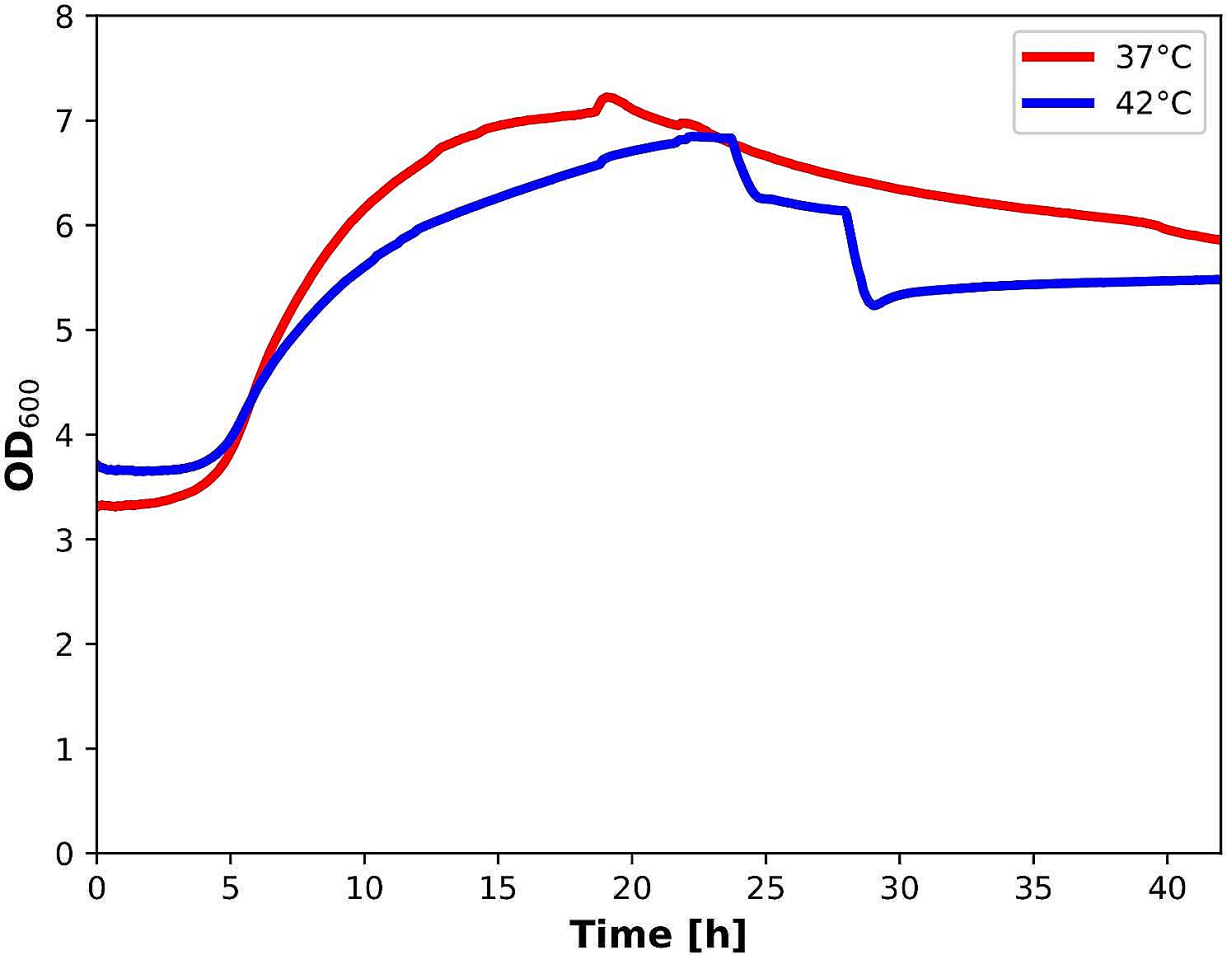
Growth curves of *C. jejuni* 81–176 recorded with the cell growth quantifier from aquila biolabs. The OD_600_ values have been deduced from the backscatter measurement and are subject to the error determined and communicated by the manufacturer in their correlation to directly measured OD_600_ values. Mass spectrometric measurements were performed at the annotated time points after 12 h and 24 h

Using the cell growth quantifier from aquila biolabs, we recorded the growth of *C. jejuni* 81–176 over 42 h. A measurement of the optical density of the bacterial suspension was taken every minute, resulting in a total of 2522 measurements per experiment. The growth curves obtained from three biological replicates at 37 °C and 42 °C are shown in Fig. 1. The optical density (OD) of the starting suspension was adjusted to an OD_600_ value of 0.5; this corresponds to an initial value of slightly above 3 in the backscatter measurement with the cell growth quantifier [17]. Both growth curves initiate with a lag phase of about four hours. The maximum exponential phase of the 37 °C curve is reached after 19 h. After that, a gradual death phase begins, which cannot be distinctively separated from a stationary phase. The exponential phase of the 42 °C curve is somewhat flatter and reaches its maximum after about 22 h. After that, we see a rather two-stage transition into the death phase. The mass spectrometric measurement point at 12 h is considered in the middle of the respective exponential phase, while the second measurement point at 24 h captures the intersection of the two growth curves. While the 42 °C curve here has reached its maximum at the end of the exponential phase, the 37 °C curve is already at the beginning of the death phase. However, when evaluating the two growth curves, it must be considered that the average standard deviation of the measurement points of the 37 °C curve is 0.27, and that of the 42 °C curve is 0.38. This means that the differences between the two growth curves are not significant at most points in time.

### Protein identification by Nano-LC-MS/MS and global proteome profiling

Using data-dependent acquisition (DDA) mass spectrometry, aliquots of each digested protein sample were analyzed to identify expressed proteins and to build a spectral library for quantitative global proteome profiling by DIA-MS. As previously published, our analysis yielded a dataset of 1079 proteins represented by 14,644 peptide sequences at 1% FDR each. Of these proteins, 1063 proved to be *C. jejuni* proteins (besides obvious contaminants like human keratin), representing 58.9% of the theoretical proteome [18]. The high peptide-to-protein ratio of 13.6 achieved here allows reliable quantification of differences in protein expression.

**Fig. 2:**
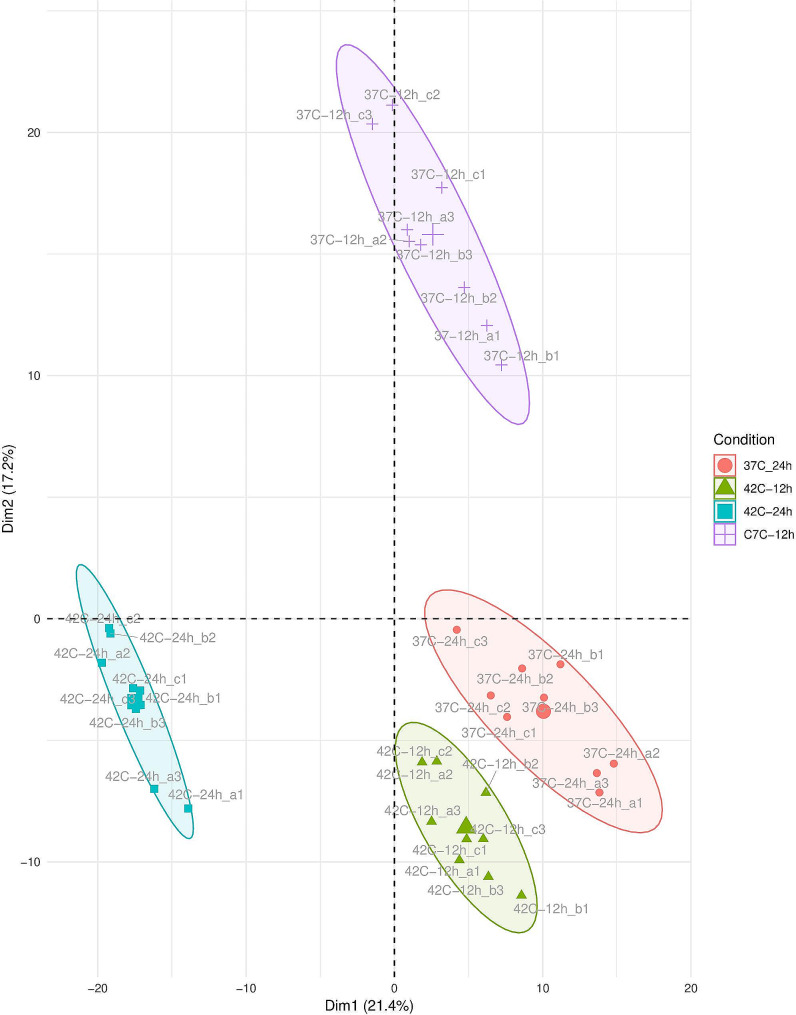
PCA analysis showing the correlation of three biological replicates measured in three technical replicates of *C. jejuni* 81–176 cultured at 37 °C and 42 °C for 12 h and 24 h

**Fig. 3:**
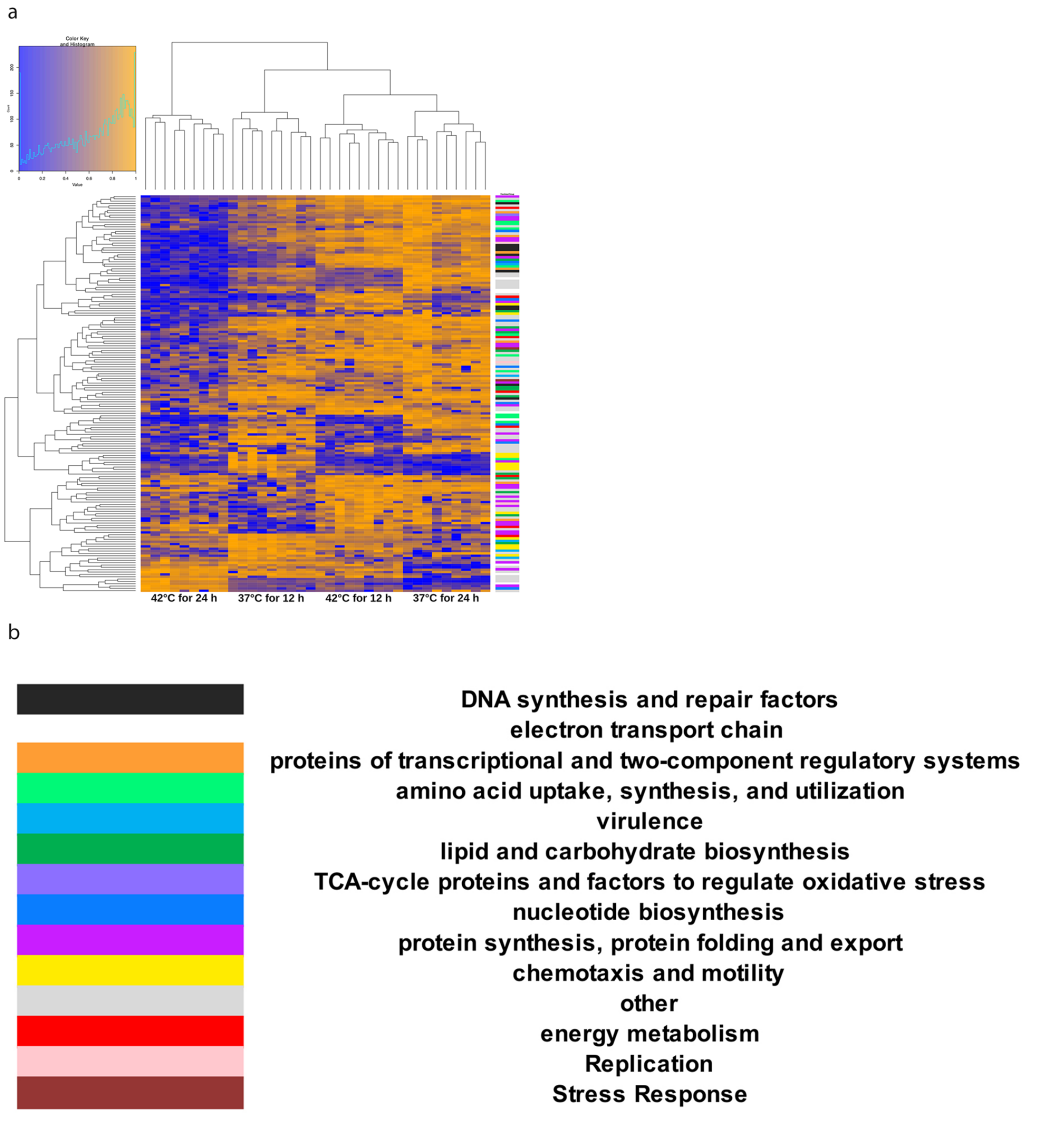
**a**. Hierarchical clustering analysis of proteins expressed at different temperatures and incubation times indicating different protein expression patterns: (1) 42 °C for 24 h, (2) 37 °C for 12 h (3) 42 °C for 12 h (4) 37 °C for 24 h. Each cluster is composed of 9 columns, resulting from three technical replicates of each of three biological replicates. **b.** Color code of the column on the right margin of Fig. 3a indicating functional groups

DIA-MS was performed for global proteome profiling. As previously reported, we were able to quantify 957 proteins (representing 53.0% of the predicted proteome) by use of a spectral library generated from the peptide-to-spectrum matches (PSMs). In total, 4298 peptides and 25 785 precursors were found to be quantifiable across all biological and technical replicates [18]. To visualize the differences between the four alternate incubation conditions and to demonstrate the reproducibility of the biological and technical replicates, a non-directed principal component analysis (PCA) was carried out. As shown in Fig. 2, all four incubation conditions can be distinguished from each other as distinct clusters. This, on the one hand, shows that the differences between the biological and technical replicates are relatively small, and thus, there is an excellent reproducibility of the methodology. On the other hand, the variabilities due to the different temperature conditions and incubation times are significantly larger than the variations due to the replicative experimental approaches and technical measurements. This, in turn, is a prerequisite for describing the biological processes at the time of measurement during the different incubation conditions. Empirical Bayesian analysis for mixed models was used to determine the *C. jejuni* 81–176 proteins detected as significantly increased or decreased at each of the four combinations of incubation time and incubation temperature (37 °C for 12 h, 37 °C for 24 h, 42 °C for 12 h, and 24 °C for 24 h). Hierarchical clustering analysis was performed with the resulting set of 170 proteins from this analysis (Fig. 3). This revealed different protein expression patterns for each of the four combinations of incubation temperature and incubation time, which in turn revealed different temperature response processes and different growth dynamics, respectively, which will now be investigated in more detail.

### Comparison of protein expression at 42 °C after 12 h and 24 h incubation

First, the proteome changes at the typical poultry body temperature of 42 °C at the incubation periods 12 h and 24 h will be described since 42 °C should represent the optimal growth conditions with respect to the avian habitat. In direct comparison of the two conditions, 65 (6.8%) proteins were detected as significantly up-regulated (log_2_ FC = > 0.585) and 336 (35.1%) as significantly down-regulated (log_2_ FC <= -0.585) after 24 h of incubation, i.e., the beginning of the stationary phase, while the expression of 556 (58.1%) proteins has not significantly changed. Conversely, this means that after 12 h, i.e., in the exponential growth phase, 336 proteins were up-regulated, and 65 proteins were down-regulated. The sheer comparison of 336 to 65 up-regulated proteins shows that significantly more metabolic systems are up-regulated in the exponential growth phase than in the stationary phase.

In order to evaluate proteome profile changes, the 957 quantifiable proteins were assigned to 16 functional categories, these were (absolute number of proteins and percentage of the 957 quantifiable proteins are given in brackets): “DNA synthesis and repair factors” (45; 4.7%), “electron transport chain” (47; 4.9%), “proteins of transcriptional and two-component regulatory systems” (12; 1.3%), “amino acid uptake, synthesis, and utilization” (99; 10.3%), “virulence” (13; 1.4%), “lipid and carbohydrate biosynthesis” (71, 7.4%), “TCA-cycle proteins and factors to regulate oxidative stress” (19, 2.0%), “nucleotide biosynthesis” (43, 4.5%), “protein synthesis, protein folding, and export” (174, 18.2%), “chemotaxis and motility” (42, 4.4%), “energy metabolism” (71, 7.4%), “replication” (20; 2.1%), “vitamin synthesis, metabolism & cofactor biosynthesis” (49; 5.1%), “DNA-Uptake” (2; 0.2%), “stress response” (18; 1.9%), and “signal transduction” (6; 0.6%). Other functions and proteins of unknown function were assigned to the category “other” (226; 23.6%).

**Fig. 4:**
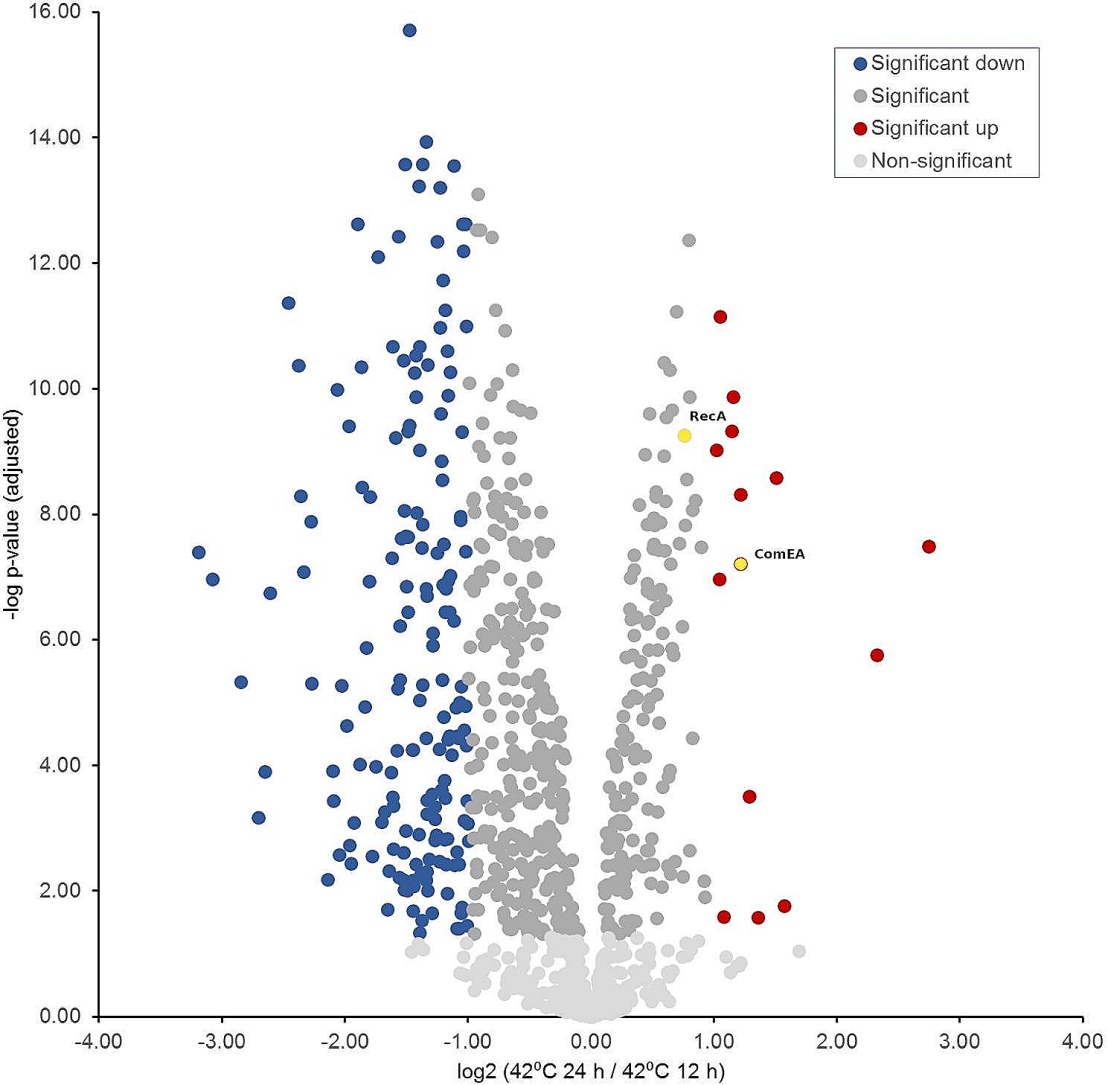
Volcano plot indicating the fold differences in protein expression levels between *C. jejuni* incubated at 42 °C for 24 h vs. 12 h. The logarithmic ratio fold change (log_2_ FC) of the protein expression difference was plotted against the negative log *p*-values. Color code: light grey: -log *p*-value < 1.30; grey, blue & red -log *p*-value > 1.30; dark red dots indicate proteins with a statistically significant up-expression (log_2_ FC = > 1) at 42 °C for 24 h (yellow dots framed red represent proteins involved in DNA uptake). Blue dots indicate proteins with a statistically significant down-expression (log_2_ FC <= -1) at 42 °C for 24 h. Dark grey dots represent proteins that show a non-significant change in expression level at both incubation times

In the pairwise comparison, more up-regulated proteins were detected in the exponential growth phase, i.e., after 12 h (Table 1; Fig. 4) in all functional categories with the only exception of the category “DNA-uptake” (0 vs. 2 proteins). Considering the percentages of the up-regulated proteins additionally in relation to the total number of proteins in each functional category, i.e., including the proteins that are not significantly differentially expressed, we recognized that mainly proteins of the categories “electron transport chain” (22/47; 46.8%), e.g. NuoM, NuoC, and Mrp, “proteins of transcriptional and two-component regulatory systems” (9/12; 75.0%), e.g. DccR, RacR, and Rrf2, “replication” (10/20; 50.0%), e.g. PbpA, ParA/B, and NapL, “virulence” (6/13; 46.2%), e.g. InvA(-like), CdtA, and, JlpA, as well as “signal transduction” (5/6; 83.3%), e.g. PDZ domain proteins and GTP-binding proteins, were relatively predominantly up-regulated. On the other hand, the two proteins, ComEA and RecA, of the functional category “DNA-uptake” (2/2; 100%) were up-regulated after 24 h of incubation compared to 12 h. The expression level of 58.1% (556/957) of the proteins did not change significantly when comparing the two-time points, 12 h vs. 24 h when incubated at 42 °C.

**Table 1:** Absolute and percentage distribution of significantly up-regulated proteins by category for protein profiles at 42 °C after 12 h and 24 h of incubation

functional category	42 °C / 12 h % of category (abs. ratio)	42 °C / 24 h % of category (abs. ratio)	Not significantly altered % of category (abs. ratio)
protein synthesis, protein folding and export	37.4 (65/174)	2.3 (4/174)	60.3 (105/174)
energy metabolism	38.0 (27/71)	2.8 (2/71)	59.2 (42/71)
electron transport chain	46.8 (22/47)	8.5 (4/47)	44.7 (21/47)
amino acid uptake, synthesis, and utilization	22.2 (22/99)	9.1 (9/99)	68.7 (68/99)
DNA synthesis and repair factors	40.0 (18/45)	4.4 (2/45)	55.6 (25/45)
chemotaxis and motility	40.5 (17/42)	7.1 (3/42)	52.4 (22/42)
vitamin synthesis, metabolism & cofactor biosynthesis	30.6 (15/49)	6.1 (3/49)	63.3 (31/49)
nucleotide biosynthesis	34.9 (15/43)	11.6 (5/43)	53.5 (23/43)
lipid and carbohydrate bio28synthesis	18.3 (13/71)	9.9 (7/71)	71.8 (51/71)
proteins of transcriptional and two-component regulatory systems	75.0 (9/12)	8.3 (1/12)	16.7 (2/12)
replication	50.0 (10/20)	0.0 (0/20)	50.0 (10/20)
stress response	38.9 (7/18)	0.0 (0/18)	61.1 (11/18)
virulence	46.2 (6/13)	23.1 (3/13)	30.8 (4/13)
TCA-cycle proteins and factors to regulate oxidative stress	26.3 (5/19	10.5 (2/19)	63.2 (12/19)
signal transduction	83.3 (5/6)	0.0 (0/6)	16.7 (1/6)
DNA-uptake	0.0 (0/2)	100 (2/2)	0.0 (0/2)
other	35.4 (80/226)	8.0 (18/226)	56.6 (128/226)
sum	35.1 (336/957)	6.8 (65/957)	58.1 (556/957)

### Comparison of protein expression at 37 °C after 12 h and 24 h incubation

**Fig. 5:**
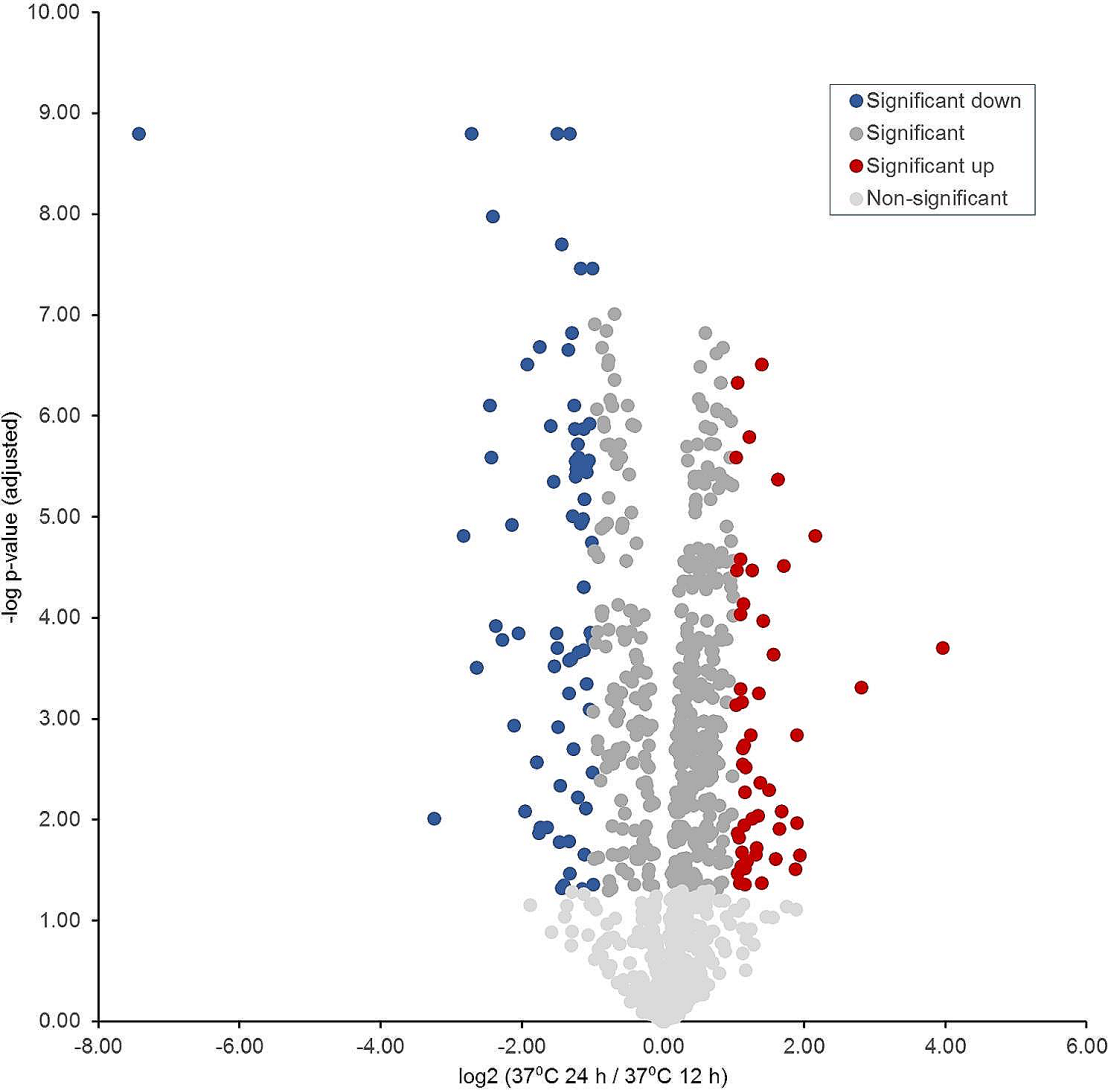
Volcano plot indicating the fold differences in protein expression levels between *C. jejuni* incubated at 37 °C for 24 h vs. 12 h. The logarithmic ratio fold change (log_2_ FC) of the protein expression difference was plotted against the negative log *p*-values. Color code: light grey: -log *p*-value < 1.30; grey, blue & red -log *p*-value > 1.30; dark red dots indicate proteins with a statistically significant up-expression (log_2_ FC = > 1) at 37 °C for 24 h. Blue dots indicate proteins with a statistically significant down-expression (log_2_ FC <= -1) at 37 °C for 24 h. Dark grey dots represent proteins that show a non-significant change in expression level at both incubation times

Second, the proteome changes at human body temperature, 37 °C, after 12 h and 24 h were compared. In a pairwise comparison of the two incubation periods at 37 °C, 193 (20.2%) proteins were detected as up-regulated (log_2_ FC = > 0.585) after 24 h, whereas 162 (16.9%) proteins were down-regulated (log_2_ FC <= -0.585). Conversely, this means that after 12 h at 37 °C, i.e., in the exponential growth phase, 162 proteins were up-regulated, while 193 proteins were down-regulated (Table 2; Fig. 5). In contrast to the comparison of the two incubation times at 42 °C, the number of differentially expressed proteins at 37 °C is relatively balanced at 193 (20.2%) to 162 (16.9%). Additionally, the expression of the majority of proteins (62.9%) was not significantly altered. This suggests that the temperature at 37 °C is a much lower stimulus for metabolism and cell division than at 42 °C. Furthermore, the significantly greater number of proteins that are up-regulated in the exponential growth phase at 42 °C compared to 37 °C (336 vs. 162) alone already indicates that metabolism and cell division are significantly more stimulated during incubation at 42 °C.

**Table 2:** Absolute and percentage distribution of significantly up-regulated proteins by category for protein profiles at 37 °C after 12 h and 24 h of incubation

functional category	37 °C / 12 h % of category (abs. ratio)	37 °C / 24 h % of category (abs. ratio)	Not significantly altered % of category (abs. ratio)
protein synthesis, protein folding and export	14.9 (26/174)	14.9 (26/174)	70.1 (122/174)
energy metabolism	19.7 (14/71)	19.7 (14/71	60.6 (43/71)
electron transport chain	40.4 (19/47)	8.5 (4/47)	51.1 (24/47)
amino acid uptake, synthesis, and utilization	11.1 (11/99)	23.2 (23/99)	65.7 (65/99)
DNA synthesis and repair factors	4.4 (2/45)	24.4 (11/45)	71.1 (32/45)
chemotaxis and motility	38.1 (16/42)	21.4 (9/42)	40.5 (17/42)
vitamin synthesis, metabolism & cofactor biosynthesis	6.1 (3/49)	38.8 (19/49)	55.1 (27/49)
nucleotide biosynthesis	0.0 (0/43)	16.3 (7/43)	83.7 (36/43)
lipid and carbohydrate biosynthesis	14.1 (10/71)	22.5 (16/71)	63.4 (45/71)
proteins of transcriptional and two-component regulatory systems	0.0 (0/12)	33.3 (4/12)	66.7 (8/12)
replication	20.0 (4/20)	20.0 (4/20	60.0 (12/20)
stress response	27.8 (5/18)	16.7 (3/18	55.6 (10/18)
virulence	38.5 (5/13)	7.7 (1/13)	53.8 (7/13)
TCA-cycle proteins and factors to regulate oxidative stress	0.0 (0/19)	10.5 (2/19)	89.5 (17/19)
signal transduction	0.0 (0/6)	33.3 (2/6)	66.7 (4/6)
DNA-uptake	50.0 (1/2)	0.0 (0/2)	50.0 (1/2)
other	20.4 (46/226)	21.2 (48/226)	58.4 (132/226)
sum	16.9 (162/957)	20.2 (193/957)	62.9 (602/957)

A pairwise comparison, 12 h vs. 24 h, of the functional categories reveals a very heterogeneous picture. Thus, after 12 h incubation, mainly proteins of the categories “electron transport chain” (19/47; 40.4%), e.g. NrfH, ChuA, and NuoC, “chemotaxis and motility” (16/42; 38.1%), e.g. flagellar proteins and chemotaxis receptors, “stress response” (5/18; 27.8%), e.g. MsrA, CmeA, and CmeA, “virulence” (5/13; 38.5%), e.g. Omp18, CadF, and CdtB, as well as “DNA-uptake” (1/2; 50.0%), e.g. ComEA, were relatively predominantly up-regulated. In contrast, after 24 h incubation at 37 °C mainly proteins of the categories “amino acid uptake, synthesis, and utilization” (23/99; 23.2%), e.g. AroQ, TyrA, and HisA, “DNA synthesis and repair factors” (11/45; 24.4%), e.g. RecJ, GyrA and components of restriction modification systems, “vitamin synthesis, metabolism & cofactor biosynthesis” (19/49; 38.8%), e.g. ThiH, FolP, and MobB, “nucleotide biosynthesis” (7/43; 16.3%), e.g. QueC, PurM, and PanB, “lipid and carbohydrate biosynthesis” (16/71; 22.5%), e.g. PglD, AccB, and PseG, “proteins of transcriptional and two-component regulatory systems” (4/12; 33.3%), e.g. SixA, RrF2, and Fur, “TCA-cycle proteins and factors to regulate oxidative stress” (2/19; 10.5%), e.g. SodB and SdhC, as well as “signal transduction” (2/6; 33.3%), e.g. GTP-binding proteins, were detected as proportionally up-regulated. Considering the percentage ratios of up-regulated proteins in the individual functional categories, i.e., including the proteins that are not significantly differentially expressed, proteins in each category are predominantly not significantly altered in their expression.

### Comparison of protein expression after 12 h incubation at 37 °C and 42 °C

**Fig. 6:**
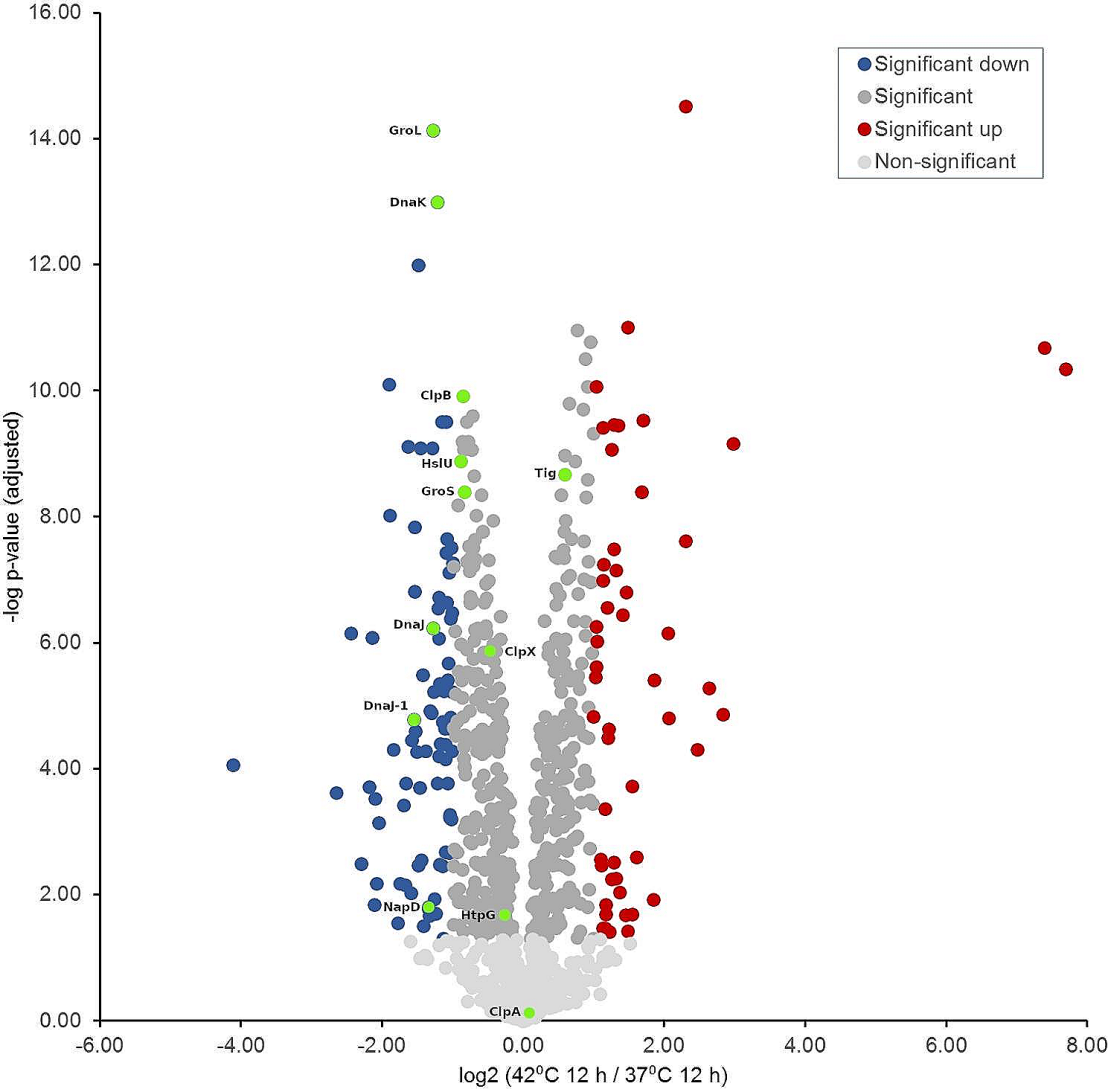
Volcano plot indicating the fold differences in protein expression levels between *C. jejuni* incubated for 12 h at 42 °C vs. 37 °C. The logarithmic ratio fold change (log_2_ FC) of the protein expression difference was plotted against the negative log *p*-values. Color code: light grey: -log *p*-value < 1.30; grey, blue & red -log *p*-value > 1.30; dark red dots indicate proteins with a statistically significant up-expression (log_2_ FC = > 1) at 42 °C for 12 h. Blue dots indicate proteins with a statistically significant down-expression (log_2_ FC <= -1) at 42 °C for 12 h (green dots represent proteins acting as chaperones). Dark grey dots represent proteins that show a non-significant change in expression level at both incubation times

Third, proteomic profiles were compared at 42 °C and 37 °C after an incubation time of 12 h (Table 3; Fig. 6). This measurement point represents the exponential growth phase under both incubation temperatures, which makes the data sets relatively well comparable. Again, the number of significant differentially expressed proteins shows a relatively balanced ratio. Thus, at 42 °C, 164 (17.1%) proteins are significantly up-regulated (log_2_ FC = > 0.585) when compared with the *C. jejuni* proteome at 37 °C. While 218 (22.8%) proteins were up-regulated when incubated at 37 °C for 12 h in comparison with the *C. jejuni* proteome at 42 °C. Similar to the other measurements, 60.1% (556/957) of the proteins are not significantly altered in their expression.

**Table 3:** Absolute and percentage distribution of significantly up-regulated proteins by category for protein profiles after 12 h of incubation at 42 °C and 37 °C

functional category	42° C / 12 h % of category (abs. ratio)	37 °C / 12 h % of category (abs. ratio)	Not significantly altered % of category (abs. ratio)
protein synthesis, protein folding and export	8.6 (15/174)	24.1 (42/174)	67.2 (117/174)
energy metabolism	12.7 (9/71)	28.2 (20/71)	59.2 (42//71)
electron transport chain	23.4 (11/47)	17.0 (8/47)	59.6 (28/47)
amino acid uptake, synthesis, and utilization	11.1 (11/99)	17.2 (17/99)	71.7 (71/99)
DNA synthesis and repair factors	17.8 (8/45)	24.4 (11/45)	57.8 (26/45)
chemotaxis and motility	31.0 (13/42)	19.0 (8/42)	50.0 (21/42)
vitamin synthesis, metabolism & cofactor biosynthesis	14.3 (7/49)	22.4 (11/49)	63.3 (31/49)
nucleotide biosynthesis	2.3 (1/43)	20.9 (9/43)	76.7 (33/43)
lipid and carbohydrate biosynthesis	18.3 (13/71)	19.7 (14/71)	62.0 (44/71)
proteins of transcriptional and two-component regulatory systems	8.3 (1/12)	50.0 (6/12)	41.7 (5/12)
replication	30.0 (6/20)	30.0 (6/20)	40.0 (8/20)
stress response	22.2 (4/18)	33.3 (6/18)	44.4 (8/18)
virulence	46.2 (6/13)	15.4 (2/13)	38.5 (5/13)
TCA-cycle proteins and factors to regulate oxidative stress	10.5 (2/19)	31.6. (6/19)	57.9 (11/19)
signal transduction	0.0 (0/6)	33.3 (2/6)	66.7 (4/6)
DNA-uptake	50.0 (1/2)	50.0 (1/2)	0.0 (0/2)
other	24.8 (56/226)	21.7 (49/226)	53.5 (121/226)
sum	17.1 (164/957)	22.8 (218/957)	60.1 (575/957)

When comparing the two conditions in pairs, it is noticeable that when incubated at 42 °C for 12 h, the proteins from only four categories: “electron transport chain” (11/47; 23.4%), e.g. hemin ABC transporter, thioredoxin, and ExbD, “chemotaxis and motility” (13/42; 31.0%), e.g. flagellar proteins and chemotaxis receptors, “virulence” (6/13; 46.2%), e.g. fibronectin type III domain protein, Omp18, and CdtB, as well as “DNA-Uptake” (1/2; 50.0%), e.g. ComEA, are proportionally more frequently up-regulated. Most notably, the category virulence is the only category that has the most up-regulated representatives at 42 °C, even taking into account the non-significantly altered expressed proteins. Virulence-associated factors represent primarily adhesion factors such as CdtB, PEB3, and Omp18, in this context. Apparently, at 42 °C, the factors for taxis to high-energy habitats, host cell, i.e., epithelial cell invasion, and provision of energy for taxis and invasion are provided to a greater extent by the microbial organism, which are indications of optimization of potential habitat evasion.

Otherwise, in the pairwise comparison at time point 12 h, all other functional categories are more frequently up-regulated in percentage at 37 °C: “protein synthesis, protein folding and export” (42/174; 24.1%), e.g. RplW, TrmB, and RpmA, “energy metabolism” (20/71; 28,2%), e.g. HypE, SdA, and CoaD, “amino acid uptake, synthesis, and utilization” (17/99; 17.2%), e.g. AroQ, HisA, and TyrA, “DNA synthesis and repair factors” (11/45; 24.4%), e.g. NrdA, GyrA, and RecJ, “vitamin synthesis, metabolism & cofactor biosynthesis” (11/49; 22.4%), e.g. MobA, ThiF, and HemC, “nucleotide biosynthesis” (9/43, 20.9%), e.g. GuaA, PurE, and PurM, “proteins of transcriptional and two-component regulatory systems” (6/12; 50.0%), e.g. SixA, RrF2, and Fur, “stress response” (6/18; 33.3%), e.g. GrpE, DedA, and TetR, “TCA-cycle proteins and factors to regulate oxidative stress” (6/19; 31.6%), e.g. IspDF, FrdB, and FrdC, “signal transduction” (2/6; 33.3%), e.g. histidine kinases, as well as DNA-uptake (1/2; 50.0%), e.g. RecA. Nevertheless, even here, only one category, “proteins of transcriptional and two-component regulatory systems,” stands out, which showed a percentual abundance even considering the proteins that are not significantly altered in their expression level. This indicates that at 37 °C the metabolic pathways such as “nucleotide biosynthesis,” “protein synthesis, protein folding and export,” “energy metabolism,” and further were additionally stimulated, which obviously occur via the up-regulation of proteins from the category “proteins of transcriptional and two-component regulatory systems.”

Of the 174 proteins in the category “protein synthesis, protein folding, and export,” 50 are ribosomal proteins, and 12 proteins function as chaperones. Within the up-regulated proteins at 37 °C, a significant number (8 of 12) of the proteins function as chaperones (GroL, DnaK, ClpB, HslU, GroS, DnaJ, DnaJ-1, NapD) are included (Fig. 6). The relative up-regulation of the proteins with chaperone function as well as the relatively more frequent up-regulation of the proteins from the category “TCA-cycle proteins and factors to regulate oxidative stress,” indicates that under incubation at 37 °C various stressors have an increased effect on the microorganism in comparison to 42 °C. Although in the overall proteome profile, after a 12 h incubation at 37 °C somewhat more proteins are up-regulated than at 42 °C, it must be noted that the number of up-regulated proteins of the category “replication” are identical with 30% each (6/20). Thus, hardly any difference in the proliferation ability can be recognized in the protein profiles after 12 h.

### Comparison of protein expression after 24 h incubation at 37 °C and 42 °C

**Fig. 7:**
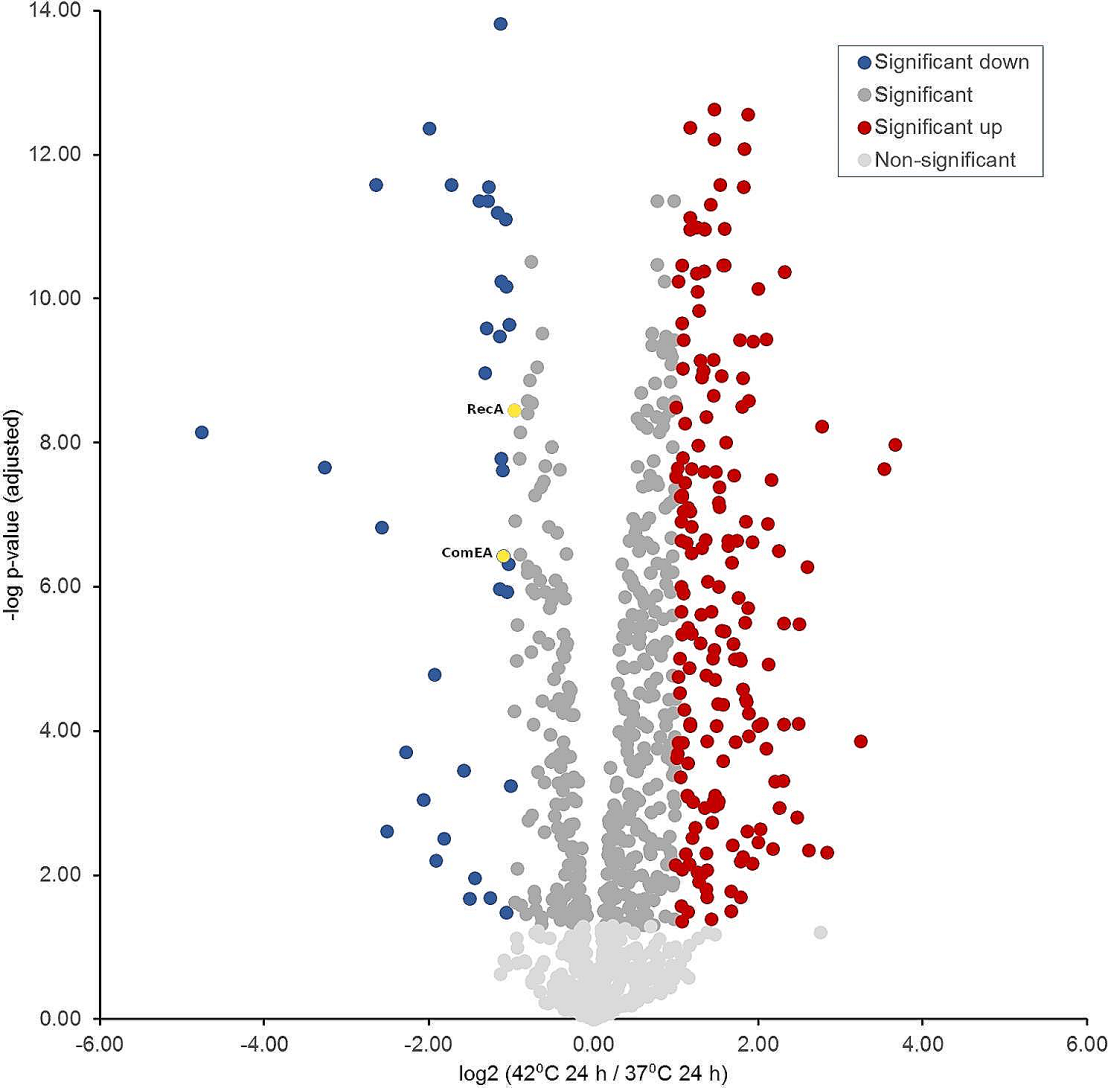
Volcano plot indicating the fold differences in protein expression levels between *C. jejuni* incubated for 24 h at 42 °C vs. 37 °C. The logarithmic ratio fold change (log_2_ FC) of the protein expression difference was plotted against the negative log *p*-values. Color code: light grey: -log *p*-value < 1.30; grey, blue & red -log *p*-value > 1.30; dark red dots indicate proteins with a statistically significant up-expression (log_2_ FC = > 1) at 42 °C for 24 h. Blue dots indicate proteins with a statistically significant down-expression (log_2_ FC <= -1) at 42 °C for 24 h (yellow dots framed blue represent proteins involved in DNA-uptake). Dark grey dots represent proteins that show a non-significant change in expression level at both incubation times

Fourth and final, the protein profiles of *C. jejuni* incubated at 42 °C and 37 °C for 24 h were compared (Table 4; Fig. 7). The time point after incubation for 24 h represents the transition from the exponential growth phase to the stationary growth phase. With regard to the number of significantly differently detected proteins, the most considerable disparity was observed here. Thus, 354 proteins were detected as significantly up-regulated when incubated at 42 °C for 24 h, compared to 99 proteins detected as significantly up-regulated when incubated at 37 °C for 24 h. At this stage of growth, it is evident that a higher incubation temperature remains a significant stimulant for most metabolic categories and cell division compared to a 12-hour incubation period. In absolute terms, proteins in all but two functional categories are significantly up-regulated in the pairwise comparison at 42 °C for 24 h. In the two categories that are exceptions, the expression levels of “TCA-cycle proteins and factors to regulate oxidative stress” remain relatively stable, with no significant change in abundance, while only the DNA-uptake category (ComEA and RecA) is more abundantly represented with two compared to zero up-regulated proteins at 37 °C compared to 42 °C.

**Table 4:** Absolute and percentage distribution of significantly up-regulated proteins by category for protein profiles after 24 h of incubation at 42 °C and 37 °C

functional category	42° C / 24 h % of category (abs. ratio)	37 °C / 24 h % of category (abs. ratio)	Not significantly altered % of category (abs. ratio)
protein synthesis, protein folding and export	32.8 (57/174)	9.8 (17/174)	57.5 (100/174)
energy metabolism	29.6 (21/71)	12.7 (9/71)	57.7 (41//71)
electron transport chain	27.7 (13/47)	17.0 (8/47)	55.3 (26/47)
amino acid uptake, synthesis, and utilization	29.3 (29/99)	8.1 (8/99)	62.6 (62/99)
DNA synthesis and repair factors	60.0 (27/45)	6.7 (3/45)	33.3 (15/45)
chemotaxis and motility	45.2 (19/42)	21.4 (9/42)	33.3 (14/42)
vitamin synthesis, metabolism & cofactor biosynthesis	42.9 (21/49)	4.1 (2/49)	63.3 (31/49)
nucleotide biosynthesis	30.2 (13/43)	9.3 (4/43)	60.5 (26/43)
lipid and carbohydrate biosynthesis	38.0 (27/71)	9.9 (7/71)	52.1 (37/71)
proteins of transcriptional and two-component regulatory systems	41.7 (5/12)	8.3 (1/12)	50.0 (6/12)
replication	50.0 (10/20)	0.0 (0/20)	50.0 (10/20)
stress response	38.9 (7/18)	11.1 (2/18)	50.0 (9/18)
virulence	30.8 (4/13)	23.1 (3/13)	46.2 (6/13)
TCA-cycle proteins and factors to regulate oxidative stress	15.8 (3/19)	15.8 (3/19)	68.4 (13/19)
signal transduction	50.0 (3/6)	0.0. (0/6)	50.0 (3/6)
DNA-uptake	0.0 (0/2)	100 (2/2)	0.0 (0/2)
other	42.0 (95/226)	9.3 (21/226)	48.7 (110/226)
sum	37.0 (354/957)	10.3 (99/957)	52.7 (504/957)

Furthermore, it is striking that in the analysis of these two conditions, the percentage of proteins not significantly altered in their expression is the lowest at 52.7%. That 42 °C represents the optimal growth temperature when compared to 37 °C is particularly evident since ten proteins from the “replication” category are up-regulated compared with zero proteins. Moreover, 27 proteins from the “DNA synthesis and repair factors” category are up-regulated compared with three proteins. In the “lipid and carbohydrate biosynthesis” category, 27 proteins are up-regulated compared to seven proteins. 21 proteins from the “vitamin synthesis, metabolism, cofactor biosynthesis” category are up-regulated compared with two proteins, and five proteins from the “proteins of transcriptional and two-component regulatory systems” category are up-regulated compared to one protein. In all these five categories, the absolute and percentage relative ratios of high-exponential proteins are particularly prominent. In the categories “amino acid uptake, synthesis, and utilization,” “nucleotide biosynthesis,” “protein synthesis, protein folding and export,” and “stress response,” the absolute number of up-regulated proteins is also higher at 42 °C compared to 37 °C.

## Discussion

Our study is the first to utilize quantitative label-free mass spectrometry (DIA-MS) for comparing the proteomic profiles of *C. jejuni* at 42 °C and 37 °C, as previous investigations have primarily relied on transcriptomic analysis [11, 12] and two-dimensional electrophoresis (2-DE) in combination with MALDI-TOF/TOF analysis [13, 14] to address this issue. The process of identifying differentially expressed proteins using 2D gel electrophoresis is distinct from the process used in DIA-MS. In 2D gel electrophoresis, proteins are separated based on their isoelectric point and molecular weight, and then identified using mass spectrometry as in Turonova et al., 2017 [14]. However, 2D gel electrophoresis has limitations, such as incomplete separation of proteins, and low sensitivity for low-abundance proteins [15, 16]. Turonova et al. identified 24 proteins while we were able to quantify 954 proteins in DIA-MS. DIA-MS, in contrast, is a method that does not require prior knowledge of the analyzed peptides. In DIA-MS, all the precursor ions within a defined m/z range are fragmented, and the resulting fragment ions are used to generate a peptide spectrum library. This library is used for identification and quantification of peptides in different samples. One advantage of DIA-MS is its ability to quantify peptides across multiple samples with high sensitivity for low-abundance proteins [19].

First, we compared the proteomic profiles at 42 °C and 37 °C over time, i.e., the logarithmic growth phase after 12 h of incubation was compared with the transition point to the stationary growth phase after 24 h. Already here, the proteins up-regulated in relation to each other showed that 42 °C is a more substantial growth stimulus than 37 °C. After 12 h incubation at 42 °C, 336 proteins were predominantly up-regulated from 15 out of 16 functional categories, whereas after 12 h incubation at 37 °C, only 162 proteins in total and mainly from five functional categories were up-regulated. When comparing the incubation times at 37 °C pairwise, the proteins in eight categories show an up-regulation after 24 h of incubation, while in the remaining categories, their expression levels are approximately equal. In the logarithmic growth phase at 42 °C, i.e., after 12 h incubation, all metabolic pathways are up-regulated with respect to anabolism, replication, and cell division, while in the stationary growth phase, we have rather a basic metabolism and partly already a stress response to toxic metabolites. In summary, the changes in the proteome profiles at 37 °C after incubation for 12 h and 24 h are similar, while at 42 °C, the 12 h incubation shows a stronger up-regulation of proteins than after 24 h.

Our analysis focuses on the comparison of the two incubation temperatures, i.e., 42 °C vs. 37 °C after 12 h and 24 h incubation. Regarding the number of differentially expressed proteins, the difference in the proteome profile after 24 h (453 differentially expressed proteins) is somewhat larger than after only 12 h incubation (382 differentially expressed proteins). It could be demonstrated that after 24 h, proteins of all categories responsible for anabolic metabolism and cell division are up-regulated at an incubation temperature of 42 °C. After 24 h incubation, the difference in proteome profiles comparing the two incubation temperatures is even more pronounced (354 vs. 99 up-regulated proteins) since 42 °C at the transition from the logarithmic to the stationary growth phase continuously represents a proliferation stimulus. Except for the proteins from the categories “virulence,” “TCA-cycle proteins and factors to regulate oxidative stress,” and “DNA uptake,” the proteins from all other functional categories are significantly up-regulated after 24 h. Although only a few proteins are generally assigned to the functional category “DNA-uptake,” such as the competence protein ComEA and the protein RecA, it should be noted that obviously, the factors for the uptake of external DNA are more strongly expressed in the stationary growth phase after incubation for ca. 24 h and at 37 °C. The uptake of external DNA can, for example, lead to the development of *C. coli*-*C. jejuni* hybrid species [20, 21]. In the synopsis of the pairwise comparisons of the two incubation temperatures after the two different incubation periods, the proteome profile changes after 12 h still prove to be very balanced, while after 24 h, there is a significantly clearer up-regulation of the proteins from the majority of the different biological processors.

The experiment carried out at an incubation time of 24 h in broth culture corresponds to the experiment performed by Zhang and coworkers using two-dimensional electrophoresis (2-DE) combined with MALDI-TOF/TOF [13]. They could detect 20 differentially expressed proteins. Comparing the incubation temperatures at 37 °C and 42 °C, 15 proteins were up-regulated, and five proteins were down-regulated at 37 °C [13]. Contrasting the results of both analyses, there is not much accordance in the differentially expressed proteins. Flavoprotein subunit of fumarate reductase (FrdA), probable thiol peroxidase (Tpx), fumarate hydratase class II (FumC), thiazole synthase (ThiG), serine hydroxymethyltransferase (GlyA), 2-oxoglutarate: acceptor oxidoreductase alpha subunit (OorA), and trigger factor (Tig), which have been detected as up-regulated at 37 °C by Zhang and coworkers, were not significantly differentially expressed in our analysis. Histidinol dehydrogenase (HisD), alkyl-hydroperoxide reductase (AphC), superoxide dismutase [Fe] (SodB), and ATP phosphoribosyltransferase (HisG) that have been demonstrated as up-regulated at 37 °C with the two-dimensional electrophoresis combined with MALDI-TOF/TOF approach, have been detected as down-regulated by DIA-MS. Only the flagellar hook protein FlgE has been detected as down-regulated at 37 °C in both analyses. To note, putative pyruvate carboxylase subunit B (Cj0933c, PaxDb: Q0P9W6), periplasmic protein p19 (Cj1659, PaxDb: Q0P7X0), bacterial non-heme ferritin (Cft, PaxDb: Q46106), two-component regulator (Cj0355c, PaxDb: Q0PBF4), putative periplasmic protein (Cj0561c, PaxDb: Q0PAV5), putative periplasmic protein (Cj1380, PaxDb: Q0P8M9), putative periplasmic protein (Cj0420, PaxDb: Q0PB90), and an outer-membrane lipoprotein carrier protein (LolA, PaxDb: Q9PNZ0) were not covered by our DIA-MS analysis.

Stintzi and coworkers used a whole genome microarray to analyze changes in transcription levels when increasing the incubation temperature of *C. jejuni* from 37 °C to 42 °C for only 50 min [12]. Thus, they only studied the temperature stress response when switching to a different incubation temperature but not the growth profile. One of the initial results of the study was that about 20% of the genes or proteins were significantly differentially expressed in their transcription level, which is notably lower compared to our study, as we detected 39.9% (382/957) of the proteins as significantly differentially expressed after 12 h and 47.3% (453/957) after 24 h. The temperature stress response detected by Stintzi et al. included a temporary down-regulation of ribosomal proteins and, thus, protein synthesis, while chaperones, chaperonins, and heat shock proteins were up-regulated [12]. If we look at our proteomic dataset, we see that after 12 h incubation, four ribosomal proteins and one chaperone were up-regulated at 42 °C while ten ribosomal proteins and eight chaperones were down-regulated; after 24 h incubation 16 ribosomal proteins and five chaperones were up-regulated at 42 °C while only six ribosomal proteins and three chaperones were down-regulated. Accordingly, the temperature stress response, indicated by the up-regulation of chaperones, chaperonins, and heat shock proteins, is overcome after 12 h at 42 °C. Conversely, the up-regulation of chaperones (8 of 12) at 37 °C can also be interpreted as a low-temperature stress response. In contrast, the down-regulation of ribosomal proteins persists after 12 h at 42 °C and then changes to an increased expression after 24 h.

Turonova et al. (2017) were able to identify 24 proteins using the 2D gel method [14]. Of these 24 proteins, 21 were also found in our dataset, with the exception of the two-component system response regulator CosR, the oxidoreductase BetA and the chaperonin GroEL. For example, *acnB*, an aconitate hydratase gene was up-regulated on a transcriptomic level in the stationary growth phase in Turonova’s study, while in our study, AcnB was not significantly up-or down-regulated at 42 °C – 37 °C. The pyridine nucleotide-disulphide oxidoreductase gene *trxB*, was significantly down-regulated on a transcriptomic level in the stationary phase in Turonova’s study, while we did not find this protein among the significantly differentially expressed proteins in none of the samples. The transcription level of *cheW* in Turonova’s study was reduced in the stationary growth phase, while in our study we found the protein up-regulated in the approach 37 °C after 12 h. The ATP-dependent chaperone ClpB found in Turonova’s work was up-regulated in our study after 12 h at 42 °C. However, Turonova’s study is barely comparable to our study, as the growth conditions and the timepoints of protein harvesting were different from ours and the proteomic methods are dissimilar.

Not entirely comparable are the studies by Konkel and colleagues and by Riedel and colleagues, who investigated a proper thermostress response at 46 °C where *Campylobacter* species can survive for only about eight hours [9, 11]. Konkel et al. were able to identify 24 proteins by analyzing a [35 S]methionine protein synthetic profile obtained by two-dimensional gel electrophoresis that were expressed preferentially in the course of a heat shock response, including specific heat shock proteins such as DnaJ, which belongs to the Hsp-40 family [9]. Riedel and colleagues used transcriptome analyses to study the thermostress response at 46 °C of *Campylobacter coli* and *Campylobacter lari*. Similar to Stintzi et al., about 17.2% (*C. coli*) or 19.4% (*C. lari*) of the genes were detected as differentially expressed, which is ca. 50% compared to our DIA-MS analysis that uses log_2_ FC = > 0.585 as threshold for significant up-regulation. In both *Campylobacter* species, the expression of the chaperone genes *clpB*, *grpE*, *dnaK*, *groEL*, *groES*, *cbpA*, and the negative transcriptional regulator *hrcA* was up-regulated after 15 min and stayed stable for one hour. Similarly, in both *Campyolabcter* species, the majority of differentially expressed proteins belonging to the functional categories “posttranslational modification, protein turn-over, and chaperones”, “coenzyme transport and metabolism” were increased in their translation, while the translation of proteins of the categories “translation, ribosomal structure and biogenesis” and “intracellular trafficking, secretion and vesicular transport” was decreased. The level of transcription of genes encoding for proteins involved in cell cycle control, cell division, chromosome partitioning or defense mechanisms stayed unaltered. This clearly shows the difference between incubation temperatures that allow optimal (42 °C) and somewhat suboptimal (37 °C) proliferation of *Campylobacter* as analyzed by us using DIA-MS, and elevated temperatures (46 °C) that represent a significant and ultimately lethal stressor.

Studies applying DIA-MS to bacteria can be broadly categorized into three groups: (A) Generating a comprehensive spectral assay library to quantify the proteome of a microbial species, (B) Analyzing a stress or response proteome in consequence to a specific stimulus compared to specific standard growth or culture conditions, and (C) Comparing proteome profiles during different growth states. Studies in category A show particularly high coverage rates of measured or quantified proteome compared to the predicted proteome based on the genome. Thus, Kusebauch and colleagues were able to detect 97% of the theoretical proteome of *Halobacterium salinarum* [22], Midha et al. detected 91.5% of the theoretical proteome of *Escheriachia coli* [23], and Malmström et al. detected 85.6% of the theoretical proteome of *Streptococcus pyogenes* [24] using DIA-MS.

In the studies of category B, we often observe a significantly lower coverage rate of the measured or quantified proteome compared to the predicted proteome. Großeholz et al. analyzed the pH adaptation of *Enterococcus faecalis* at approximately 53% coverage rate [25], and in a recent study of our group examining the bile acid response of *E. faecalis* and *Enterococcus faecium* the coverage rates were 43.5% and 45.8% [26], while Kang et al. studied the stress response of Methicillin-resistant *Staphylococcus aureus* to lactobionic acid at approximately 47% coverage rate [27]. Furthermore, other studies targeting specific protein fractions, such as membrane proteins or cytosolic proteins, using different pre-separation methods, cannot be compared here as the coverage is inherently less than 10% due to the method used. Such studies include the investigation of the proteome of carbapenemase-producing *E. coli* under carbapenem exposure [28], the proteome of *Salmonella enterica* under thymol exposure [29], the proteome of *Clostridium cellulovorans* under butanol stress [30], or the proteome of *Pseudomonas aeruginosa* under naphthalene exposure [31]. While Chen et al. achieved a coverage of approximately 35% in their analysis of the proteome profiles of *C. jejuni* under peracetic acid exposure [32], the coverage was approximately 61% in the analysis of deoxycholate-induced stress by Man et al. [33]. Our analysis, with a coverage of 58.9%, shows a similarly high coverage [18], which we were able to increase to 83.6% using a modernized technique [34].

In category C, we find, among others, the proteomic analysis of spiral and coccoid forms of *Helicobacter pylori* with a coverage of approximately 61% [35]. Additionally, there is the proteome comparison of *Pseudomonas aeruginosa* in biofilm versus planktonic bacterial cells, with a coverage of approximately 39% [36]. Lastly, there is the analysis of proteomic profiles of *E. coli* under various growth conditions, including nutrient limitations, non-metabolic stresses, and non-planktonic states, with a coverage of approximately 64% [37]. Currently, a coverage of around 60% of the theoretical proteome is considered standard, which could be increased to 80–90% in the near future with the help of better mass spectrometers.

## Conclusions

DIA-MS is an appropriate method to characterize proteome profiles of *C. jejuni* at different incubation temperatures and different incubation times. In contrast to two-dimensional electrophoresis (2-DE) combined with MALDI-TOF/TOF, significantly more proteins can be identified, and their expression level quantified by DIA-MS. Thus, this method is roughly equivalent to transcriptome analysis depending on the selected threshold for a differential expression or transcription level. Incubation at 42 °C, the body temperature of birds, the natural habitat of *C. jejuni*, is a significantly stronger stimulus of replication and an anabolic metabolism compared to 37 °C. This was indicated by the up-regulation of proteins of the functional categories “replication”, e.g. PbpA, ParA, ParB, and NapL, “proteins of transcriptional and two-component regulatory systems”, e.g. DccR, RacR, and Rrf2, “electron transport chain”, e.g. NuoM, NuoC, and Mrp, as well as “virulence” (6/13; 46.2%), e.g. InvA(-like), CdtA, and, JlpA, during the exponential growth phase at 42 °C. However, the factors for competence to uptake extraneous DNA, ComEA and RecA, are up-regulated at an incubation temperature of 37 °C for 24 h. The up-regulation of the chaperones GroL, DnaK, ClpB, HslU, GroS, DnaJ, DnaJ-1, and NapD at 37 °C in comparison to 42 °C after 12 h incubation indicates a temporary lower-temperature response. However, further studies are needed to validate the use of DIA-MS as a method to characterize stress responses and adaptational processes of bacteria, and suitable analysis and visualization methods must be applied to functionally classify the different expression levels of the proteins.

## Methods

### Growth of *Campylobacter jejuni* 81–176 at different temperatures

*C. jejuni* 81–176 was purchased from the American Type Culture Collection (ATCC-BAA-2151, lot/batch No. 70,020,897). This strain was chosen for our proteomic experiments due to the availability of an annotated and reviewed proteome (https://www.uniprot.org/taxonomy/354242). Additionally, the strain’s multilocus sequencetype ST-42 and the genetic markers *ansB*, *dmsA*, and *ggt*, indicative of an extended amino acid metabolism, pointing to a primary avian host, most likely chickens, for this isolate [38]. *C. jejuni* 81–176 was grown at 42 °C in *Campylobacter*-defined broth (CDB) as described previously [18] for 16 h under microaerophilic conditions (85% N_2_, 10% CO_2_, 5% O_2_; using CampyGen™ sachets in a AnaeroJar™; Oxoid), shaking at 150 rpm. After incubating the culture for 16 h, the OD_600_ reached approximately 1, which allows for a straightforward dilution to a value of 0.5. At this time point, the bacterial cells are still in the exponential growth phase. To prepare for the next experimental step, the optical density was adjusted to an OD_600_ of 0.5. Subsequently, 10 mL of the cultures were transferred into 150 mL Erlenmeyer flasks and incubated under microaerophilic conditions for 42 h in technical triplicates at 37 °C and 42 °C, shaking at 150 rpm. After 42 h of incubaton, it is highly likely that the stationary growth phase has been surpassed, and the process of bacterial cell death in the culture has begun. Parallelized real-time growth monitoring was performed in at three different ocassions (biological triplicates) by the cell growth quantifier (CGQ) from aquila biolabs (aquila biolabs GmbH, Baesweiler, Germany).

### Sample preparation for DIA analysis

The sample preparation was carried out as previously described [18]. In brief, *C. jejuni* 81–176 was cultured in CDB for 12 h (a time point approximately in the middle of the exponential growth phase) and 24 h (a time point after the begin of the stationary phase) at 37 °C and 42 °C under shaking at 150 rpm. Suspension cultures were harvested by centrifugation at 3,500xg for ten minutes, and the obtained bacterial pellets were resuspended in cooled 1 mL of physiological saline solution. The bacteria in the suspension were disrupted using a digital sonifier model 250 (Branson Ultrasonic Corporation, Danbury, Connecticut, USA); Configuration: 5 bursts at a setting of 3 and 30% duty cycles for 30 s with 30 s intervals. Cell debris was removed by centrifuging at 4 °C and 15,300xg for 15 min. Using SDS-PAGE (4–12% gradient tris Newpage Gel; Invitrogen/Thermo Fisher Scientific, Waltham, Massachusetts, USA; Ref Nr. NP0321, Log Nr. 22,091,610), the harvested proteins were separated for ca. 5 min (samples run ca. 1.5 cm into the gel). Visualization was done by Colloidal Coomassie staining. Protein bands were excised and diced. This was followed by digestion with trypsin overnight. The tryptic peptides were extracted from the gel, and the solution was then dried in a Speedvac and stored at -20 °C for further analysis [39]. Quantification of the protein concentration was performed using the Pierce BCA Protein Assay Kit (λ = 562 nm; Thermo Scientific, Rockford, USA). The extracted proteins were purified using acetone precipitation at -20 °C overnight with acetone: sample proportion of 4:1. The protein pellets were washed with ice-cold acetone and subsequently air-dried. Using sodium 3-[(2-methyl-2-undecyl-1, 3-dioxolan-4-yl)-methoxy]-1-propanesulfonate (Rapigest, Waters, Eschborn, Germany) trypsin cleavable surfactant, the pellets were redissolved. Digestion of proteins was performed using sequencing grade porcine trypsin (Promega, Mannheim, Germany) at a 1:50 enzyme: substrate ratio (w: w) after the reduction and alkylation of cysteine residues using dithiothreitol and iodoacetamide. Afterwards, acidic cleavage of the surfactant was performed. The remaining fatty acids were removed by centrifugation. The obtained peptides were concentrated in a SpeedVac Concentrator centrifuge (Thermo Scientific, Darmstadt, Germany) and finally stored at -20 °C prior to analysis.

### LC-MS/MS data acquisition

Using a Nanoflow chromatography system (Eksigent nanoLC425, SCIEX, Darmstadt, Germany) connected to a hybrid triple quadrupole-time of flight mass spectrometer (TripleTOF 5600+, SCIEX, Darmstadt, Germany), equipped with a Nanospray III ion source (Ionspray Voltage 2200 V, Interface Heater Temperature 150 °C, Sheath Gas Setting 10) and controlled by Analyst TF 1.6 software (all AB Sciex, Darmstadt, Germany), protein digests were analyzed. Peptides from each digestion process were dissolved using loading buffer (2% aqueous acetonitrile vs. 0.1% formic acid) until a concentration of 0.5 µg/µL was reached and subsequently desalted using a trap column (Dr. Maisch, Ammerbuch-Entringen, Germany; RP-C18aq, particle size 5 μm, 30 × 0.150 mm, 60 µL loading buffer). Separation was performed by reversed phase-C18 nanoflow chromatography (Dr. Maisch, Ammerbuch-Entringen, Germany; RP-C18aq, particle size 3 μm, 250 × 0.075 mm, linear gradient 90 min 5% > 35% acetonitrile vs. 0.1% formic acid, 300 nL/min, 50 °C). The following qualitative LC-MS/MS analysis was performed with a Top25 data-dependent acquisition (DDA) method using an MS survey scan of m/z 380–1250 accumulated for 250 ms at a resolution of 35,000 FWHM (Full Width at Half Maximum). For 100 ms, MS/MS scans of m/z 180–1750 were accumulated at a resolution of 17,500 FWHM and a precursor isolation width of 0.7 FWHM, which resulted in a total cycle time of 3.4 s. For MS/MS, precursors with a threshold intensity over 200 cps with charge states 2+, 3+, and 4 + were chosen with a dynamic exclusion time of 15 s. For qualitative analysis, identification of proteins, and the generation of a spectral library for targeted data extraction, two technical replicates of each sample containing 1.5 µg protein were used. Data-independent acquisition (DIA) analysis was performed, and MS/MS data were acquired for 100 precursor segments of adjustable size (5–40 Th), resulting in a precursor m/z range of 400–1250. Production of fragments was done by rolling collision energy settings, and fragments were obtained over an m/z range of 380–1600 for an accumulation time of 40 ms per segment. This included a 250 ms survey scan and resulted in a cycle time of 4.5 s. For quantitative analysis, three technical replicates containing 2.0 µg protein of each sample were acquired.

### LC-MS/MS data processing

Identification of proteins was accomplished using ProteinPilot V5.0 (AB Sciex, Darmstadt, Germany) at “thorough” settings. The 551.443 MS/MS spectra from the previous combined qualitative analyses were compared to the *C. jejuni* 81–176 proteome from UniProtKB with 1804 protein entries (https://www.uniprot.org/dataset/identifier) complemented with 51 frequently occurring lab and workflow contaminants. Using forward/reverse decoy database approach, the global false discovery rates (FDR) were calibrated to 1% at the protein and the peptide level. Utilization of the DIA quantitation microApp V2.0 enabled DIA peak extraction in PeakView V2.1 (AB Sciex, Darmstadt, Germany). After the retention time alignment to a set of 12 endogenous peptides, the peak areas were isolated. An extracting ion current (XIC) with a width of 75 ppm and an XIC window of 8 min was used for the eight highest-scoring peptides at six transitions per peptide in a protein group, and filtering to an estimated FDR of 1% was done. After retention time alignment on a set of 12 endogenous peptides, peak areas were extracted for up to the eight highest scoring peptides per protein group at six transitions per peptide, an extracting ion current (XIC) width of 75 ppm, and an XIC window of 8 min, and filtered to an estimated FDR of 1%. Mass spectrometry data were deposited at the ProteomeXchange Consortium via the PRIDE [40] partner repository with the dataset identifier PXD029494.

### Statistical and bioinformatics analysis

For further statistical analyses, the acquired peak areas were exported at the fragment, peptide, and protein levels. To determine significantly up-or down-regulated proteins of *C. jejuni* 81–176 in the different temperature conditions, the Empirical Bayes method for mixed models implemented in the R Bioconductor limma package was utilized [41, 42]. UniProt accessions were replaced with gene names prior to analysis. Mixed model analysis was then performed in two steps. In the first step, the regression coefficient of the effect of each of the four temperature/time period combinations on the expression of each gene was independently calculated. In the second step, the regression coefficients of each combination of incubation temperature and incubation time were compared in a single equation to establish a relationship regarding the influence of protein expression and incubation conditions. In the final step, moderated t-statistics were used to determine the changes in protein expression between different incubation temperatures/times. Proteins that exhibited a 1.5-fold change FC > 1.5 or FC<-1.5 (i.e., log_2_ FC > 0.585 or log_2_ FC < -0.585 and an FDR-adjusted *p* value less than 0.05 were considered to have significant differential expression.

### Electronic supplementary material

Below is the link to the electronic supplementary material.

Supplementary Material 1: Figure S1: High resolution version of Figure 3

Supplementary Material 2: Table S1: Differencially expressed proteins according to Temperature

## Data Availability

Mass spectrometry data were deposited at the ProteomeXchange Consortium via the PRIDE (Vizcaíno et al., 2014) partner repository with the dataset identifier PXD029494.

## Abbreviations

CDB *Campylobacter*: defined broth
CGQ: cell growth quantifier
cps: counts per second
FDR: false discovery rate
FWHM: full width at half maximum
DDA: data-dependent acquisition
DIA: data-INdependent acquisition
LC: liquid chromatography
log< Subscript>2</Subscript> FC: natural logarithm of a number to base-2 of fold-change
MALDI: Matrix-assisted Laser Desorption/Ionization
MS: mass spectrometry
OD: optical density
PCA: principal component analysis
PCR: polymerase chain reaction
PSMs: peptide-to-spectrum matches
TCA: tricarboxylic acid cycle
TOF: time of flight
XIC: extracting ion current
2-DE: two-dimensional electrophoresis

## References

[1] Alter T, Bereswill S, Glunder G, Haag LM, Hänel I, Heimesaat MM (2011). [Campylobacteriosis of man: livestock as reservoir for *Campylobacter* species]. Bundesgesundheitsblatt Gesundheitsforschung Gesundheitsschutz.

[2] Kaakoush NO, Castaño-Rodríguez N, Mitchell HM, Man SM (2015). Global Epidemiology of *Campylobacter* Infection. Clin Microbiol Rev.

[3] European Food Safety Authority, European Centre for Disease Prevention and Control. The European Union summary report on trends and sources of zoonoses, zoonotic agents and food-borne outbreaks in 2016. EFSA J. 2017;15. 10.2903/j.efsa.2017.5077 PMC7009962 32625371

[4] Awad WA, Hess C, Hess M (2018). Re-thinking the chicken-*Campylobacter jejuni* interaction: a review. Avian Pathol J WVPA.

[5] Kist M, Bereswill S (2001). Campylobacter jejuni. Contrib Microbiol.

[6] Crushell E, Harty S, Sharif F, Bourke B (2004). Enteric Campylobacter: Purging Its Secrets? Pediatr Res.

[7] Zautner AE, Johann C, Strubel A, Busse C, Tareen AM, Masanta WO (2014). Seroprevalence of campylobacteriosis and relevant post-infectious sequelae. Eur J Clin Microbiol Infect Dis.

[8] Lecuit M, Abachin E, Martin A, Poyart C, Pochart P, Suarez F (2004). Immunoproliferative small intestinal disease associated with *Campylobacter jejuni*. N Engl J Med.

[9] Konkel ME, Kim BJ, Klena JD, Young CR, Ziprin R (1998). Characterization of the thermal stress response of *Campylobacter jejuni*. Infect Immun.

[10] Bhaduri S, Cottrell B (2004). Survival of Cold-stressed *Campylobacter jejuni* on Ground Chicken and Chicken skin during Frozen Storage. Appl Environ Microbiol.

[11] Riedel C, Förstner KU, Püning C, Alter T, Sharma CM, Gölz G. Differences in the Transcriptomic Response of *Campylobacter coli* and *Campylobacter lari* to heat stress. Front Microbiol. 2020;11. 10.3389/fmicb.2020.00523 PMC7118207 32292399

[12] Stintzi A (2003). Gene expression Profile of *Campylobacter jejuni* in response to growth temperature variation. J Bacteriol.

[13] Zhang M-J, Xiao D, Zhao F, Gu Y-X, Meng F-L, He L-H (2009). Comparative proteomic analysis of *Campylobacter jejuni* cultured at 37°C and 42°C. Jpn J Infect Dis.

[14] Turonova H, Haddad N, Hernould M, Chevret D, Pazlarova J, Tresse O. Profiling of *Campylobacter jejuni* Proteome in Exponential and Stationary Phase of Growth. Front Microbiol. 2017;8. 10.3389/fmicb.2017.00913 PMC5435804 28572800

[15] Huang Q, Yang L, Luo J, Guo L, Wang Z, Yang X (2015). SWATH enables precise label-free quantification on proteome scale. Proteomics.

[16] Van den Bergh G, Arckens L (2005). Recent advances in 2D electrophoresis: an array of possibilities. Expert Rev Proteom.

[17] AquilaBiolabs., AQUILA BIOLABS CGQ USER MANUAL Pdf Download. ManualsLib. https://www.manualslib.com/manual/2515287/Aquila-Biolabs-Cgq.html. Accessed 1 Dec 2023.

[18] Masanta WO, Zautner AE, Lugert R, Bohne W, Gross U, Leha A et al. Proteome profiling by label-free Mass Spectrometry reveals differentiated response of *Campylobacter jejuni* 81–176 to sublethal concentrations of bile acids. Proteom Clin Appl. 2018;:e1800083. 10.1002/prca.201800083 PMC6585709 30246935

[19] Birk T, Wik MT, Lametsch R, Knøchel S (2012). Acid stress response and protein induction in *Campylobacter jejuni* isolates with different acid tolerance. BMC Microbiol.

[20] Golz JC, Epping L, Knüver M-T, Borowiak M, Hartkopf F, Deneke C (2020). Whole genome sequencing reveals extended natural transformation in *Campylobacter* impacting diagnostics and the pathogens adaptive potential. Sci Rep.

[21] Dieckmann A-L, Riedel T, Bunk B, Spröer C, Overmann J, Groß U et al. Genome and methylome analysis of a phylogenetic novel *Campylobacter coli* cluster with *C. jejuni* introgression. Microb Genomics. 2021;7. 10.1099/mgen.0.000679 PMC8627207 34661518

[22] Kusebauch U, Lorenzetti APR, Campbell DS, Pan M, Shteynberg D, Kapil C (2023). A comprehensive spectral assay library to quantify the Halobacterium salinarum NRC-1 proteome by DIA/SWATH-MS. Sci Data.

[23] Midha MK, Kusebauch U, Shteynberg D, Kapil C, Bader SL, Reddy PJ (2020). A comprehensive spectral assay library to quantify the *Escherichia coli* proteome by DIA/SWATH-MS. Sci Data.

[24] Malmström L, Bakochi A, Svensson G, Kilsgård O, Lantz H, Petersson AC (2015). Quantitative proteogenomics of human pathogens using DIA-MS. J Proteom.

[25] Großeholz R, Koh C-C, Veith N, Fiedler T, Strauss M, Olivier B (2016). Integrating highly quantitative proteomics and genome-scale metabolic modeling to study pH adaptation in the human pathogen *Enterococcus faecalis*. Npj Syst Biol Appl.

[26] Dreyer A, Lenz C, Groß U, Bohne W, Zautner AE (2024). Comparative analysis of proteomic adaptations in *Enterococcus faecalis* and *Enterococcus faecium* after long term bile acid exposure. BMC Microbiol.

[27] Kang S, Kong F, Liang X, Li M, Yang N, Cao X (2019). Label-free quantitative proteomics reveals the Multitargeted Antibacterial mechanisms of Lactobionic Acid against Methicillin-Resistant *Staphylococcus aureus* (MRSA) using SWATH-MS technology. J Agric Food Chem.

[28] Sidjabat HE, Gien J, Kvaskoff D, Ashman K, Vaswani K, Reed S (2018). The use of SWATH to analyse the dynamic changes of bacterial proteome of carbapanemase-producing *Escherichia coli* under antibiotic pressure. Sci Rep.

[29] Qi Y, Zhao W, Wang T, Pei F, Yue M, Li F (2020). Proteomic analysis of the antimicrobial effects of sublethal concentrations of thymol on *Salmonella enterica* Serovar Typhimurium. Appl Microbiol Biotechnol.

[30] Costa P, Usai G, Re A, Manfredi M, Mannino G, Bertea CM (2021). Clostridium cellulovorans proteomic responses to Butanol stress. Front Microbiol.

[31] Imam A, Suman SK, Singh P, Vempatapu BP, Tripathi D, Ray A (2023). Proteomic response of *Pseudomonas aeruginosa* IIPIS-8 during rapid and efficient degradation of naphthalene. Environ Res.

[32] Chen SH, Bose U, Broadbent JA, Fegan N, Wilson R, Kocharunchitt C (2023). Proteome analysis of *Campylobacter jejuni* poultry strain 2704 survival during 45 min exposure to peracetic acid. Int J Food Microbiol.

[33] Man L, Dale AL, Klare WP, Cain JA, Sumer-Bayraktar Z, Niewold P (2020). Proteomics of *Campylobacter jejuni* growth in deoxycholate reveals Cj0025c as a cystine transport protein required for wild-type human infection phenotypes. Mol Cell Proteom MCP.

[34] Dreyer A, Lenz C, Groß U, Bohne W, Zautner AE (2023). Characterization of *Campylobacter jejuni* proteome profiles in co-incubation scenarios. Front Microbiol.

[35] Loke MF, Ng CG, Vilashni Y, Lim J, Ho B (2016). Understanding the dimorphic lifestyles of human gastric pathogen *Helicobacter pylori* using the SWATH-based proteomics approach. Sci Rep.

[36] Erdmann J, Thöming JG, Pohl S, Pich A, Lenz C, Häussler S (2019). The core proteome of biofilm-grown clinical *Pseudomonas aeruginosa* isolates. Cells.

[37] Mori M, Zhang Z, Banaei-Esfahani A, Lalanne J-B, Okano H, Collins BC (2021). From coarse to fine: the absolute *Escherichia coli* proteome under diverse growth conditions. Mol Syst Biol.

[38] Zautner AE, Herrmann S, Corso J, Tareen AM, Alter T, Groß U (2011). Epidemiological association of different *Campylobacter jejuni* groups with metabolism-associated genetic markers. Appl Env Microbiol.

[39] Atanassov I, Urlaub H (2013). Increased proteome coverage by combining PAGE and peptide isoelectric focusing: comparative study of gel-based separation approaches. Proteomics.

[40] Vizcaíno JA, Deutsch EW, Wang R, Csordas A, Reisinger F, Ríos D (2014). ProteomeXchange provides globally coordinated proteomics data submission and dissemination. Nat Biotechnol.

[41] Ritchie ME, Phipson B, Wu D, Hu Y, Law CW, Shi W (2015). *Limma* powers differential expression analyses for RNA-sequencing and microarray studies. Nucleic Acids Res.

[42] Smyth GK (2004). Linear models and empirical bayes methods for assessing differential expression in microarray experiments. Stat Appl Genet Mol Biol.

