# Supplementary figures and images for: Proteome profiling of Campylobacter jejuni 81–176 at 37 °C and 42 °C by label-free mass spectrometry
Source: BMC Microbiol. 2024 May 31;24:191. doi: 10.1186/s12866-024-03348-8 (PMC11140963; doi:10.1186/s12866-024-03348-8)

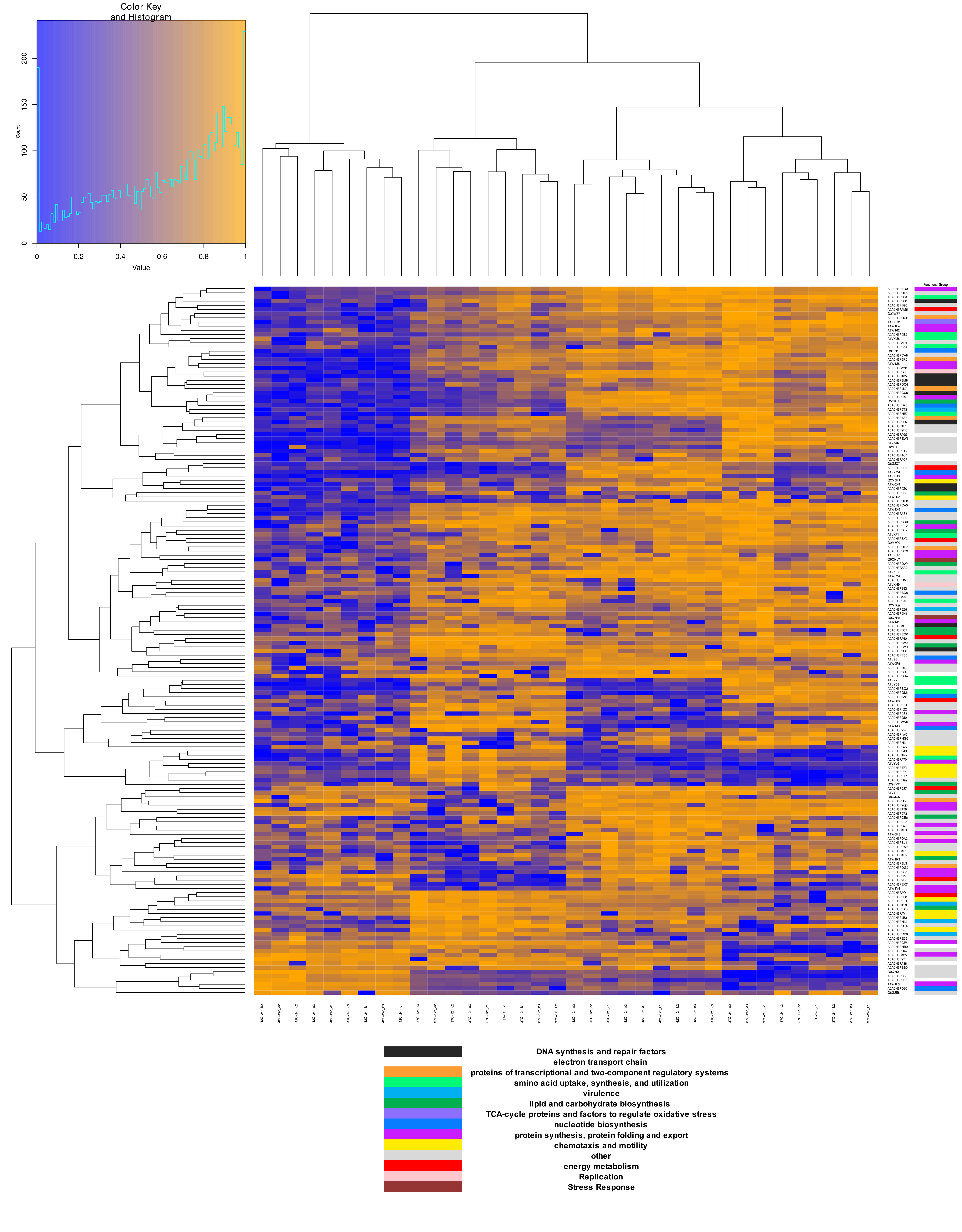

Supplement: Supplementary file 1 — Supplementary Material 1: Figure S1: High resolution version of Figure 3 [file 12866_2024_3348_MOESM1_ESM.jpg]
